# Supplementary material for: From Spaceflight to Mars g-Levels: Adaptive Response of A. Thaliana Seedlings in a Reduced Gravity Environment Is Enhanced by Red-Light Photostimulation
Source: Int J Mol Sci. 2021 Jan 18;22(2):899. doi: 10.3390/ijms22020899 (PMC7830483; doi:10.3390/ijms22020899)
Supplement: Supplementary file 1 [file ijms-22-00899-s001.pdf]

Supplementary Material

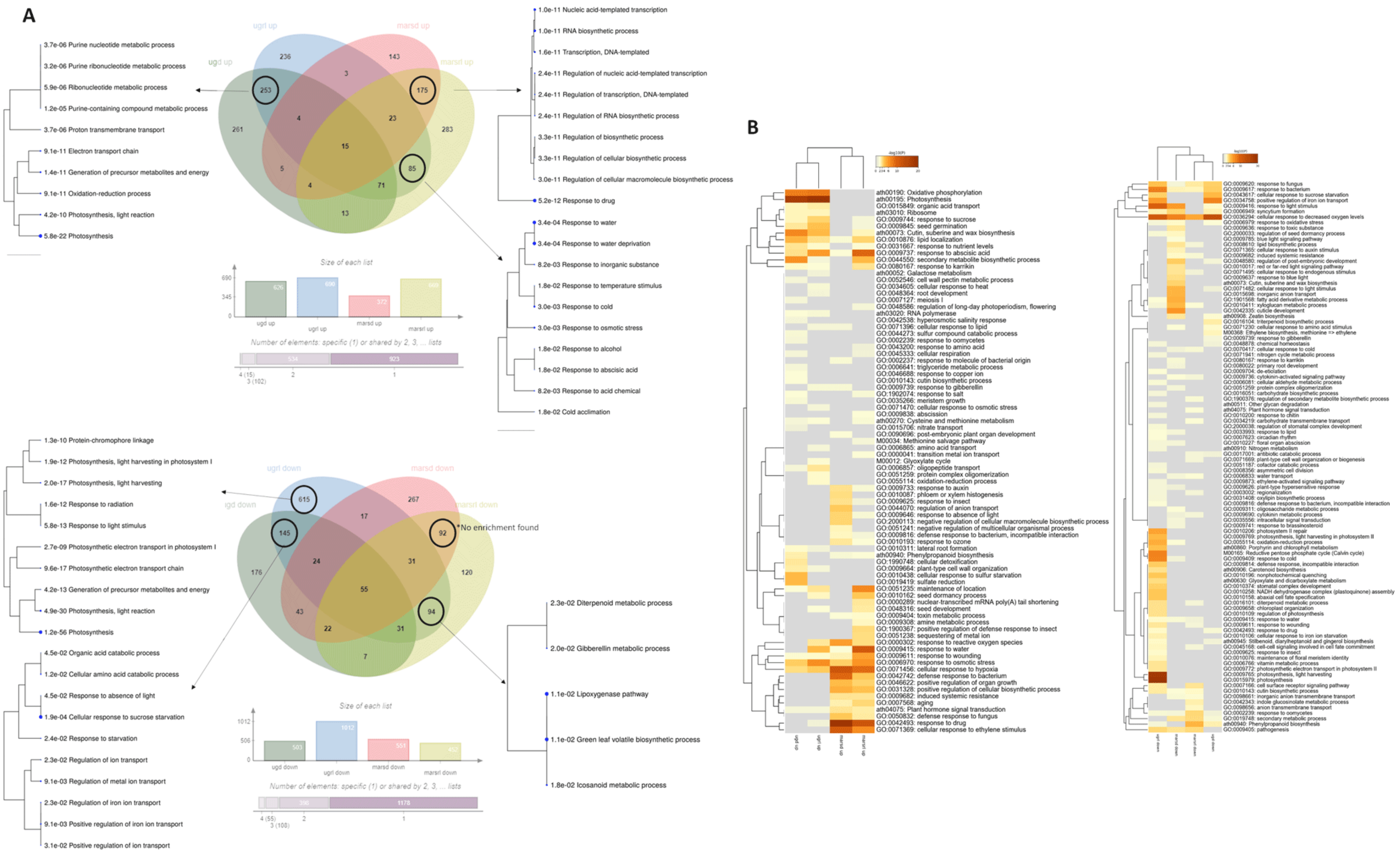

Figure S1. Extended Gene Ontology clusters affected by microgravity or Mars gravity. A) Venn diagrams with up (up) and down (down) DEG with GO Biological process analysis of specific sublists. B) Extended Heatmaps of Gene Ontology Analysis. Top 100 enriched clusters are shown for up and downregulated genes in the comparisons: µgd-grrd, µgrl-grrrl, marsd-grrd and marsrl-grrrl.

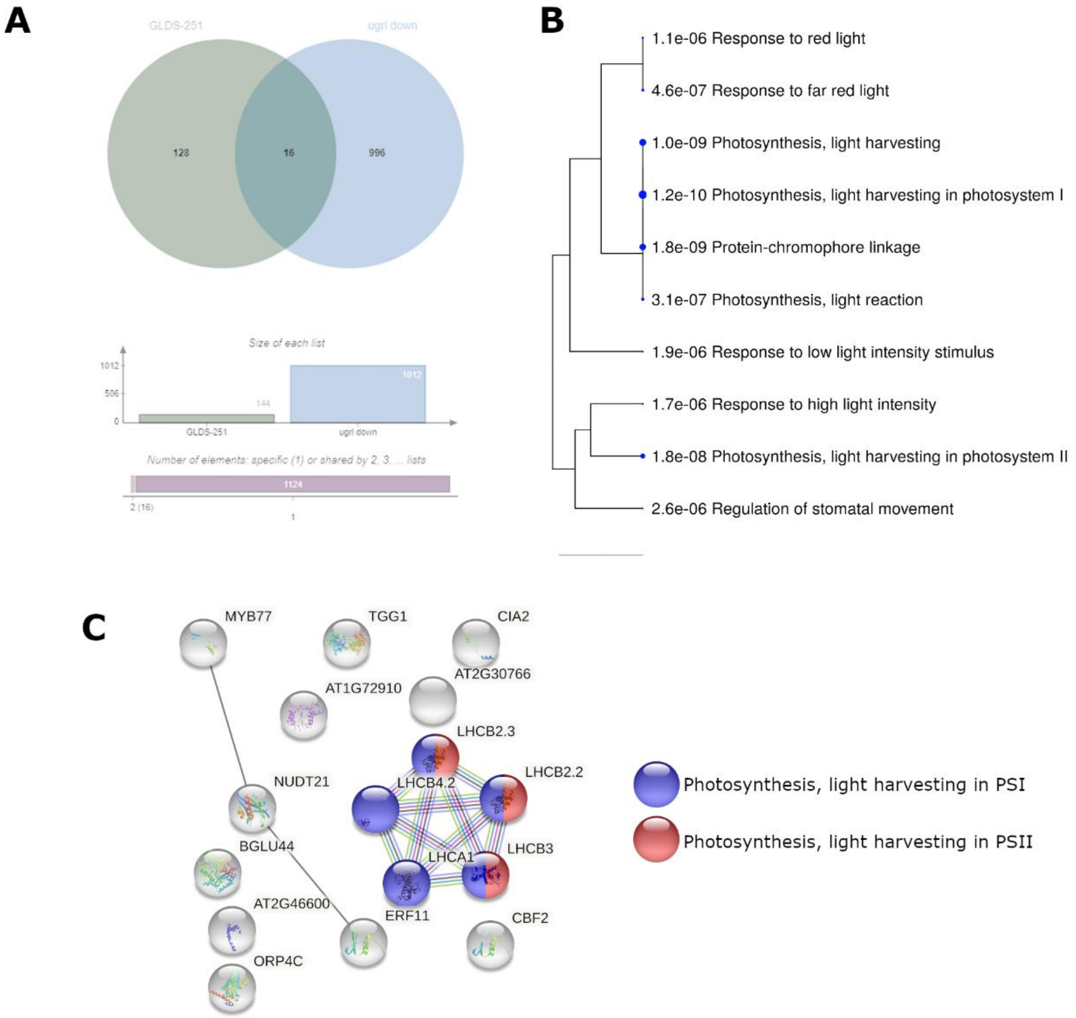

Figure S2. Common microgravity downregulated photosynthesis genes in GLDS-314 (µgrl-grrrl comparison, Red photostimulated) and GLDS-251 (Blue photostimulated). A) Venn diagram showing the overlap in the downregulated genes in microgravity from the two studies. B) Biological process Gene Ontology analysis of the 16 common genes. C) Representation of the 16 common genes with String v.11, where PPI are shown. Genes highlighted in blue represent PSI related genes, red: PSII related genes.

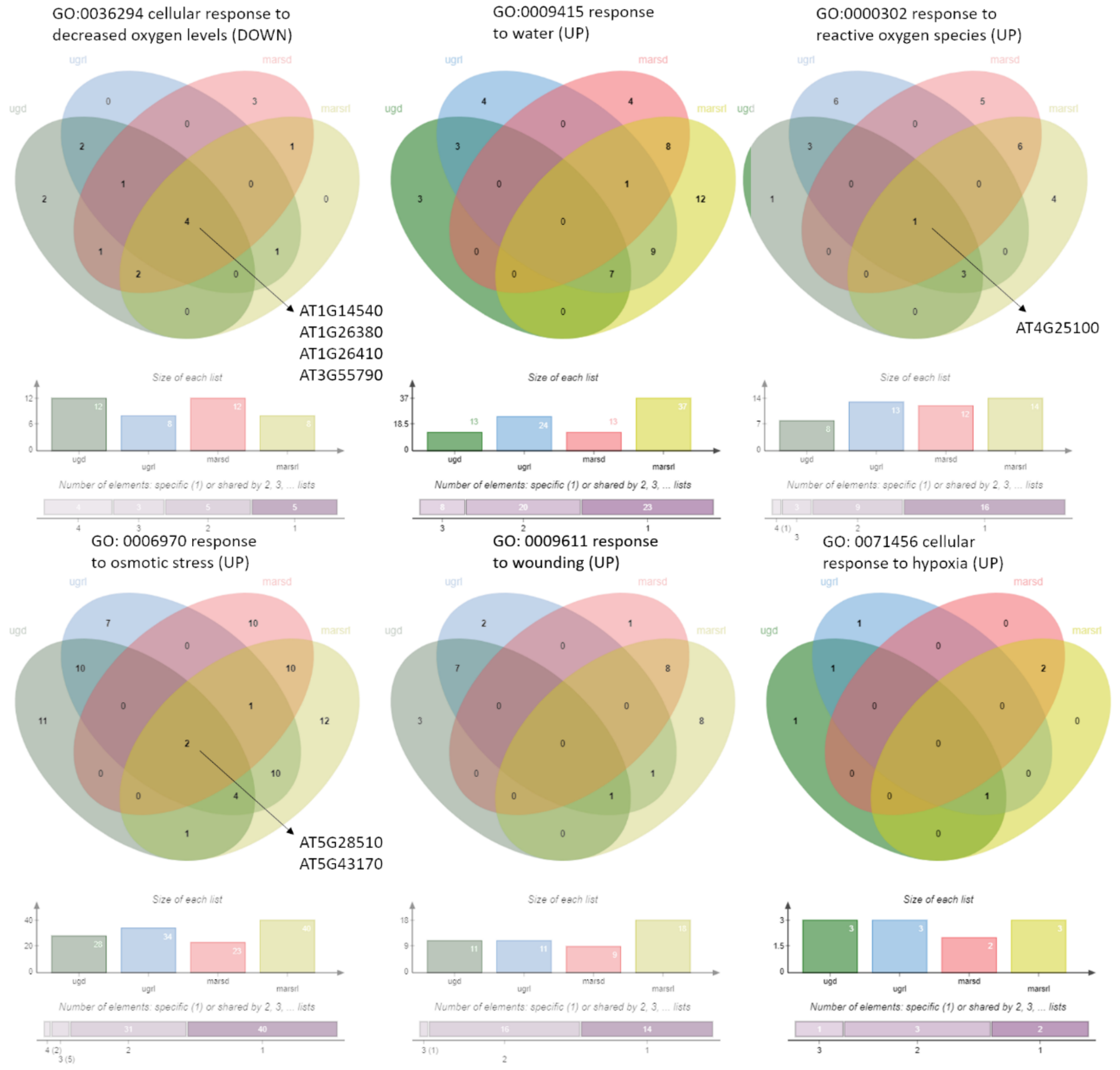

Figure S3. Venn diagrams of DEG in common GO categories to the four comparisons: µgd-grrd, µgrl-grrrl, marsd-grrd and marsrl-grrrl.

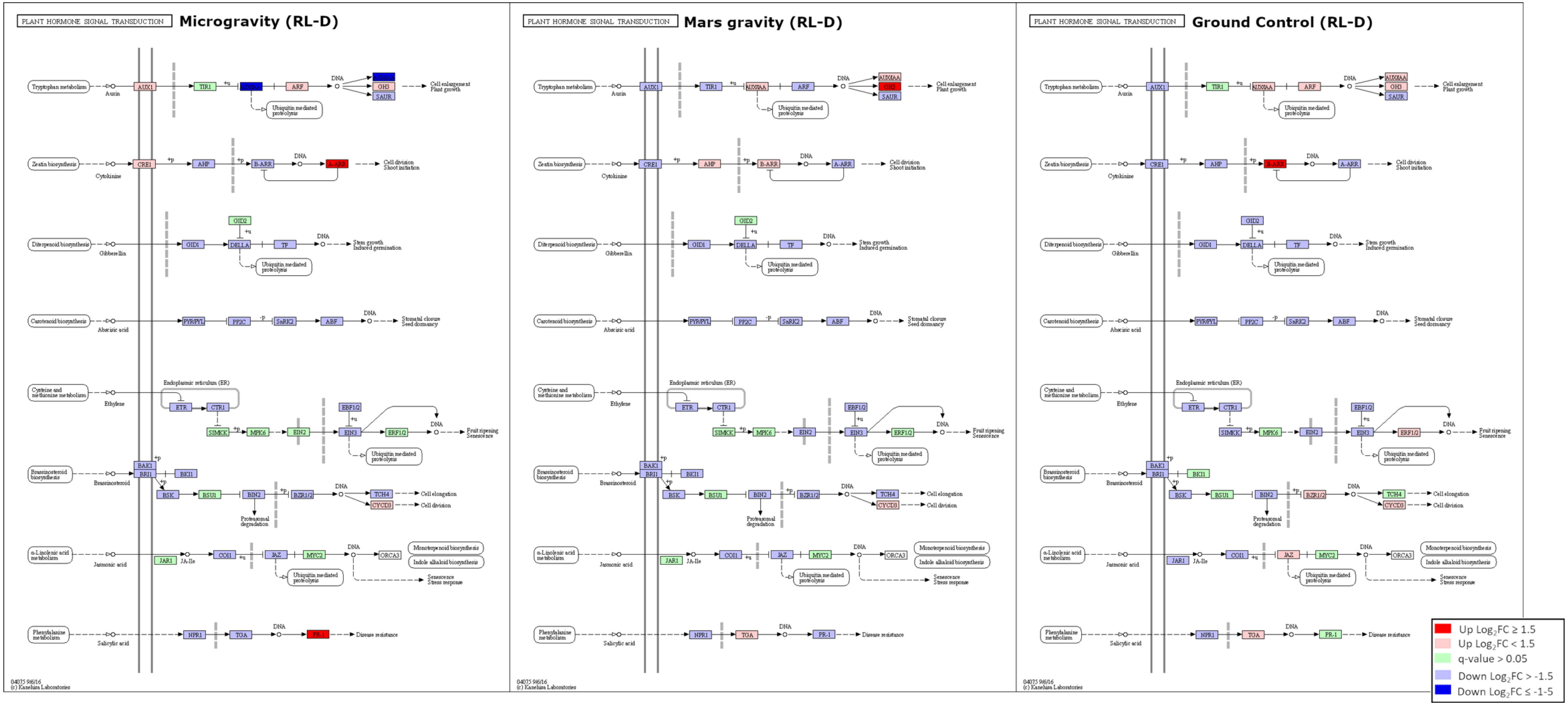

Figure S4. Hormone signal transduction changes in red light compared to darkness. KEGG ath04075 pathway.

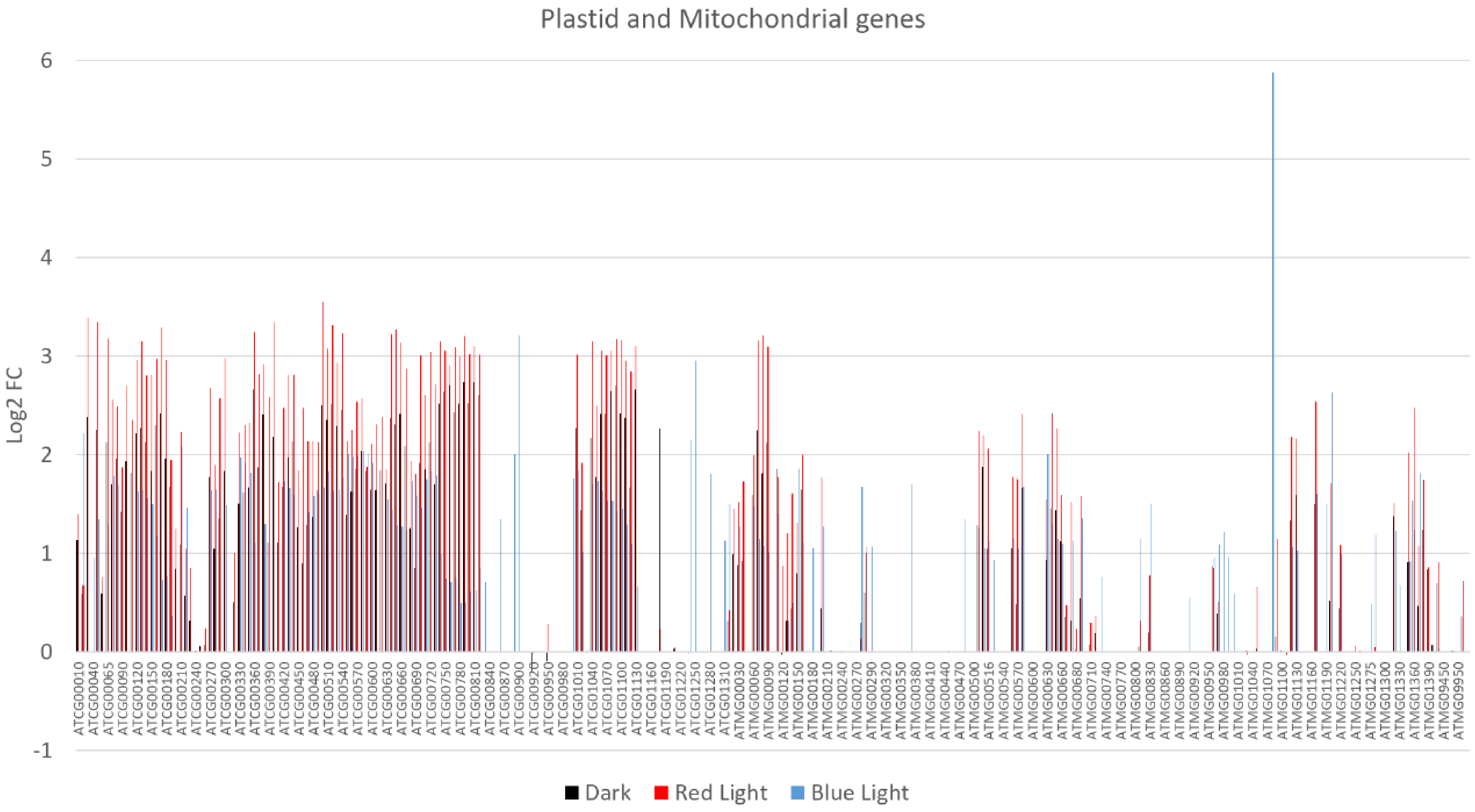

Figure S5. Plastid and Mitochondrial genome expression. Log_2FC representation of all plastid and mitochondrial genome expression in three comparisons: µgd-grrd, µgrl-grrrl and GLDS-251 blue light stimulated seedlings (microgravity compared to 1*g* control).

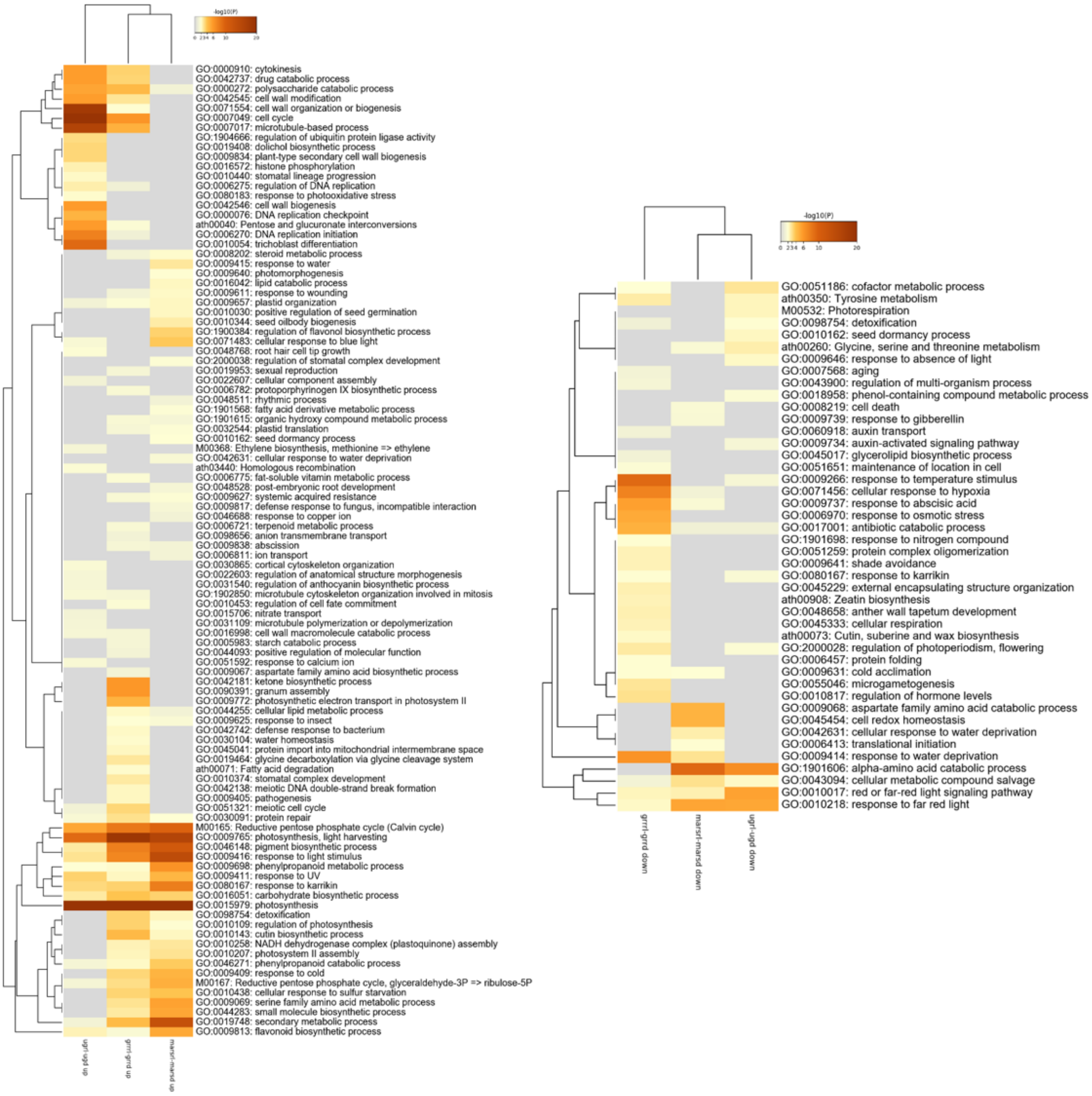

Figure S6. Extended heatmaps of Gene Ontology Analysis of the effect of red light photostimulation across gravity levels. Top 100 (or total number) of enriched clusters for down and upregulated genes in the comparisons: ugrl-ugd, marsrl-marsd and grrrl-grrd.

Supplementary table 1. List of the genes and the Log_2 FC values of the dysregulated in microgravity (μgd_grrd, μgrl_grrrl comparisons). Bold values are statistically significant (q – value < 0.05).

Name	ID	μgd_grrd Log_2FC	μgrl_grrrl Log_2FC
AHP3	AT5G39340	**0.48**	**0.43**
AHP4	AT3G16360	**2.59**	**1.69**
PIF4	AT2G43010	**-1.16**	**-1.72**
PIL6	AT3G59060	**-1.16**	**-0.83**
HAI2	AT1G07430	0.81	**2.03**
HAI3	AT2G29380	**1.76**	**3.25**
BSU1	AT1G03445	-0.57	**-1.99**
TCH4	AT5G57560	**-1.18**	**-1.65**
JAZ4	AT1G48500	**2.85**	**3.06**
AIB	AT2G46510	**-1.56**	**-1.45**
PR1-like	AT2G19990	**-2.69**	**-3.18**
-	AT1G50060	**-2.22**	**1.06**
TG	AT1G36060	**-1.72**	**-0.78**
CBF1	AT4G25490	**-3.52**	**-3.2**
CBF2	AT4G25470	**-4.18**	**-3.53**
ERF72	AT3G16770	**-1.3**	**-1.91**
ANAC017	AT1G34190	**-0.4**	**-0.47**
SIB1	AT3G56710	**-1.04**	-0.55
WRKY45	AT3G01970	**-0.92**	**-1.12**

Supplementary table 2. List of the genes and the Log_2 FC values of the dysregulated in Mars gravity (marsd_grrd. marsdrl_grrrl comparisons). Bold values are statistically significant (q – value < 0.05).

Name	ID	marsd_grrd	marsrl_grrrl
HK2	AT5G35750	**-1.67**	**-0.97**
HAI2	AT1G07430	**2.06**	**2.84**
HAI3	AT2G29380	**2.38**	**4.02**
SNRK2.9	AT2G23030	**1.54**	**0.90**
SNRK2.5	AT5G63650	**1.32**	**0.85**
ERF1	AT3G23240	**3.21**	**3.01**
ERF2	AT5G47220	**3.94**	**2.84**
BSU1	AT1G03445	**-1.78**	**-1.95**
TCH4	AT5G57560	**-0.93**	**-1.45**
JAZ1	AT1G19180	**1.67**	**1.81**
-	AT4G33720	0.46	**2.89**
SIB1	AT3G56710	**2.53**	**1.67**
WRKY45	AT3G01970	**1.2**	**0.81**
OM66	AT3G50930	**2.58**	**3.27**
WRKY40	AT1G80840	**2.13**	**1.82**
DIC1	AT2G22500	**1.39**	**1.78**
DIC2	AT4G24570	**2.07**	**1.49**

Supplementary table 3. Upregulated pastid and mitochondrial genes in microgravity dark (compared to grrd). microgravity red light (compared to grrl) and the Genelab databases: GLDS-251 (in microgravity). GLDS-38 and GLDS-44. ✓: gene upregulated in the indicated dataset. -: gene not upregulated.

Gene ID	Microgravity Dark	Microgravity RL	GLDS-251 Microgravity	GLDS-38	GLDS-44
**ATCG00010**	-	-	-	✓	-
**ATCG00020**	-	-	✓	-	✓
**ATCG00030**	✓	✓	-	-	-
**ATCG00050**	✓	✓	-	-	✓
**ATCG00065**	✓	✓	-	-	✓
**ATCG00070**	✓	✓	✓	-	✓
**ATCG00080**	✓	✓	✓	-	✓
**ATCG00090**	-	✓	-	✓	-
**ATCG00100**	✓	✓	-	-	-
**ATCG00110**	✓	✓	-	-	-
**ATCG00120**	✓	✓	✓	-	✓
**ATCG00130**	✓	✓	✓	-	✓
**ATCG00140**	✓	✓	-	-	✓
**ATCG00150**	✓	✓	✓	-	✓
**ATCG00160**	✓	✓	-	-	-
**ATCG00170**	✓	✓	-	-	✓
**ATCG00180**	✓	✓	-	-	-
**ATCG00190**	✓	✓	-	-	✓
**ATCG00210**	-	✓	✓	-	✓
**ATCG00220**	-	-	✓	-	-
**ATCG00230**	-	-	-	✓	-
**ATCG00270**	✓	✓	✓	✓	✓
**ATCG00280**	-	✓	-	-	✓
**ATCG00290**	-	✓	-	✓	-
**ATCG00300**	✓	✓	-	-	✓
**ATCG00310**	-	-	-	✓	-
**ATCG00320**	-	-	-	✓	-
**ATCG00330**	✓	✓	✓	-	✓
**ATCG00340**	✓	✓	✓	-	-
**ATCG00350**	✓	✓	✓	-	✓
**ATCG00360**	✓	✓	-	-	✓
**ATCG00370**	✓	✓	-	✓	-
**ATCG00380**	✓	✓	-	✓	-
**ATCG00390**	-	✓	-	-	-
**ATCG00400**	✓	✓	-	-	-
**ATCG00410**	-	✓	-	-	-
**ATCG00420**	✓	✓	✓	-	✓
**ATCG00430**	✓	✓	✓	-	✓
**ATCG00440**	✓	✓	✓	-	-
**ATCG00450**	-	✓	-	-	-

**ATCG00460**	-	√	-	√	-
**ATCG00470**	-	√	√	-	-
**ATCG00480**	-	√	√	-	-
**ATCG00490**	√	√	-	-	√
**ATCG00500**	√	√	√	√	√
**ATCG00510**	√	√	√	-	-
**ATCG00520**	√	√	√	√	√
**ATCG00530**	√	√	√	√	√
**ATCG00540**	√	√	√	√	-
**ATCG00550**	-	√	√	√	-
**ATCG00560**	√	√	√	-	-
**ATCG00570**	√	√	√	√	-
**ATCG00580**	√	√	√	-	-
**ATCG00590**	√	√	√	-	√
**ATCG00600**	√	√	√	√	√
**ATCG00610**	√	√	-	√	-
**ATCG00620**	√	√	-	√	-
**ATCG00630**	√	√	√	-	√
**ATCG00640**	√	√	-	√	√
**ATCG00650**	√	√	-	-	√
**ATCG00660**	√	√	-	-	√
**ATCG00670**	√	√	-	-	-
**ATCG00680**	-	√	√	-	√
**ATCG00690**	-	√	√	-	√
**ATCG00700**	√	√	-	-	√
**ATCG00710**	√	√	√	-	√
**ATCG00720**	√	√	√	-	-
**ATCG00730**	√	√	√	√	√
**ATCG00740**	√	√	-	-	-
**ATCG00750**	√	√	-	-	-
**ATCG00760**	√	√	-	√	-
**ATCG00770**	√	√	-	√	√
**ATCG00780**	√	√	-	-	-
**ATCG00790**	√	√	-	-	-
**ATCG00800**	√	√	-	-	-
**ATCG00810**	√	√	-	-	-
**ATCG00820**	√	√	-	-	-
**ATCG00830**	-	-	-	√	-
**ATCG00840**	-	-	-	√	-
**ATCG00850**	-	-	-	√	-
**ATCG00860**	-	-	-	√	-
**ATCG00870**	-	-	-	√	-
**ATCG00880**	-	-	-	√	-
**ATCG00890**	-	-	-	√	-
**ATCG00900**	-	-	√	√	-

**ATCG00905**	-	-	-	√	-
**ATCG00910**	-	-	-	√	-
**ATCG00920**	-	-	-	√	-
**ATCG00930**	-	-	-	√	-
**ATCG00940**	-	-	-	√	-
**ATCG00950**	-	-	-	√	-
**ATCG00960**	-	-	-	√	-
**ATCG00970**	-	-	-	√	-
**ATCG00980**	-	-	-	√	-
**ATCG00990**	-	-	-	√	-
**ATCG01000**	-	-	√	√	-
**ATCG01010**	√	√	√	-	√
**ATCG01020**	-	√	√	-	√
**ATCG01030**	-	-	-	√	-
**ATCG01040**	√	√	√	-	√
**ATCG01050**	√	√	√	√	√
**ATCG01060**	√	√	√	-	√
**ATCG01070**	√	√	√	-	√
**ATCG01080**	√	√	√	-	√
**ATCG01090**	√	√	√	-	-
**ATCG01100**	√	√	-	-	-
**ATCG01110**	√	√	-	-	√
**ATCG01120**	√	√	-	√	√
**ATCG01130**	√	√	-	-	-
**ATCG01140**	-	-	-	√	-
**ATCG01150**	-	-	-	√	-
**ATCG01160**	-	-	-	√	-
**ATCG01170**	-	-	-	√	-
**ATCG01180**	√	-	-	√	-
**ATCG01190**	-	-	-	√	-
**ATCG01200**	-	-	-	√	-
**ATCG01210**	-	-	-	√	-
**ATCG01220**	-	-	-	√	-
**ATCG01230**	-	-	-	√	-
**ATCG01240**	-	-	-	√	-
**ATCG01250**	-	-	√	√	-
**ATCG01300**	-	-	-	√	-
**ATMG00030**	-	√	-	-	-
**ATMG00040**	-	√	-	-	-
**ATMG00060**	√	√	-	√	-
**ATMG00070**	√	√	-	√	-
**ATMG00080**	√	√	-	-	-
**ATMG00090**	√	√	-	-	-
**ATMG00100**	-	-	-	√	-
**ATMG00110**	√	√	-	-	-

**ATMG00120**	-	-	-	√	-
**ATMG00130**	-	-	-	√	-
**ATMG00140**	-	√	-	√	-
**ATMG00150**	-	-	-	√	-
**ATMG00160**	√	√	-	-	-
**ATMG00170**	-	-	-	√	-
**ATMG00180**	-	-	-	√	-
**ATMG00190**	-	-	-	√	-
**ATMG00200**	-	√	-	√	-
**ATMG00210**	-	-	-	√	-
**ATMG00220**	-	-	-	√	-
**ATMG00230**	-	-	-	√	-
**ATMG00240**	-	-	-	√	-
**ATMG00250**	-	-	-	√	-
**ATMG00260**	-	-	-	√	-
**ATMG00270**	-	-	-	√	-
**ATMG00290**	-	-	-	√	-
**ATMG00420**	-	-	-	√	-
**ATMG00430**	-	-	-	√	-
**ATMG00440**	-	-	-	√	-
**ATMG00460**	-	-	-	√	-
**ATMG00470**	-	-	-	√	-
**ATMG00510**	-	√	-	√	-
**ATMG00513**	√	√	-	√	-
**ATMG00516**	-	√	-	√	-
**ATMG00520**	-	-	-	√	-
**ATMG00530**	-	-	-	√	-
**ATMG00540**	-	-	-	√	-
**ATMG00560**	-	√	-	-	-
**ATMG00570**	-	√	-	√	-
**ATMG00580**	√	√	-	-	-
**ATMG00590**	-	-	-	√	-
**ATMG00600**	-	-	-	√	-
**ATMG00610**	-	-	-	√	-
**ATMG00620**	-	-	-	√	-
**ATMG00630**	-	√	-	√	-
**ATMG00640**	-	√	-	-	-
**ATMG00650**	-	√	-	-	-
**ATMG00660**	-	√	-	-	-
**ATMG00670**	-	√	-	-	-
**ATMG00680**	-	-	-	√	-
**ATMG00690**	-	√	√	-	-
**ATMG00710**	-	-	-	√	-
**ATMG00720**	-	-	-	√	-
**ATMG00810**	-	-	-	-	√

**ATMG00880**	-	-	-	-	√
**ATMG00890**	-	-	-	√	-
**ATMG00910**	-	-	-	√	-
**ATMG00960**	-	-	-	√	-
**ATMG00970**	-	-	-	√	-
**ATMG00980**	-	-	√	-	-
**ATMG00990**	-	-	√	-	-
**ATMG01080**	-	-	√	-	-
**ATMG01100**	-	-	-	√	-
**ATMG01110**	-	-	-	√	-
**ATMG01120**	-	√	-	-	-
**ATMG01130**	√	√	-	-	-
**ATMG01170**	-	√	-	-	-
**ATMG01190**	-	-	-	√	-
**ATMG01200**	-	√	√	-	-
**ATMG01250**	-	-	-	√	√
**ATMG01290**	-	-	-	-	√
**ATMG01320**	-	√	-	-	-
**ATMG01350**	-	√	-	-	-
**ATMG01360**	√	√	-	-	-
**ATMG01380**	-	√	-	√	-

Table 1 References list

1. Kim KC, Lai Z, Fan B, Chen Z. *Arabidopsis* WRKY38 and WRKY62 transcription factors interact with histone deacetylase 19 in basal defense. *Plant Cell* **2008**, *20*, 2357–71.
2. Pandey SP, Roccaro M, Schön M, Logemann E, Somssich IE. Transcriptional reprogramming regulated by WRKY18 and WRKY40 facilitates powdery mildew infection of *Arabidopsis*. *Plant J* **2010**, *64*, 912–23.
3. Finatto T, Viana VE, Woyann LG, Busanello C, Maia LC da, Oliveira AC de. Can WRKY transcription factors help plants to overcome environmental challenges? *Genet Mol Biol* **2018**, *41*, 533–44.
4. van Aken O, de Clercq I, Ivanova A, Law SR, van Breusegem F, Millar AH, et al. Mitochondrial and chloroplast stress responses are modulated in distinct touch and chemical inhibition phases. *Plant Physiol* **2016**, *171*, 2150–65.
5. Meng X, Li L, Narsai R, De Clercq I, Whelan J, Berkowitz O. Mitochondrial signalling is critical for acclimation and adaptation to flooding in *Arabidopsis thaliana*. *Plant J* **2020**, *103*, 227–47.
6. Wang H, Xu Q, Kong YH, Chen Y, Duan JY, Wu WH, et al. *Arabidopsis* WRKY45 transcription factor activates Phosphate transporter1;1 expression in response to phosphate starvation. *Plant Physiol* **2014**, *164*, 2020–9.
7. Ding ZJ, Yan JY, Li CX, Li GX, Wu YR, Zheng SJ. Transcription factor WRKY46 modulates the development of *Arabidopsis* lateral roots in osmotic/salt stress conditions via regulation of ABA signaling and auxin homeostasis. *Plant J* **2015**, *84*, 56–69.
8. Hu Y, Dong Q, Yu D. *Arabidopsis* WRKY46 coordinates with WRKY70 and

WRKY53 in basal resistance against pathogen Pseudomonas syringae. *Plant Sci* **2012**, *185–186*, 288–97.

9. Lee TA, Bailey-Serres J. Integrative analysis from the epigenome to translatome uncovers patterns of dominant nuclear regulation during transient stress. *Plant Cell* **2019**, *31*, 2573–95.
10. Gao QM, Venugopal S, Navarre D, Kachroo A. Low oleic acid-derived repression of jasmonic acid-inducible defense responses requires the WRKY50 and WRKY51 proteins. *Plant Physiol* **2011**, *155*, 464–76.
11. Miao Y, Zentgraf U. A HECT E3 ubiquitin ligase negatively regulates *Arabidopsis* leaf senescence through degradation of the transcription factor WRKY53. *Plant J* **2010**, *63*, 179–88.
12. Besseau S, Li J, Palva ET. WRKY54 and WRKY70 co-operate as negative regulators of leaf senescence in *Arabidopsis thaliana*. *J Exp Bot* **2012**, *63*, 2667–79.
13. Chen L, Song Y, Li S, Zhang L, Zou C, Yu D. The role of WRKY transcription factors in plant abiotic stresses. *Biochim Biophys Acta - Gene Regul Mech* **2012**, *1819*, 120–8.
14. Li Z, Peng J, Wen X, Guo H. Gene network analysis and functional studies of senescence-associated genes reveal novel regulators of *Arabidopsis* leaf senescence. *J Integr Plant Biol* **2012**, *54*, 526–39.
15. Jiang Y, Yu D. The WRKY57 transcription factor affects the expression of Jasmonate ZIM-domain genes transcriptionally to compromise *Botrytis cinerea* resistance. *Plant Physiol* **2016**, *171*, 2771–82.
